# Efficient Stereo Depth Estimation for Pseudo-LiDAR: A Self-Supervised Approach Based on Multi-Input ResNet Encoder

**DOI:** 10.3390/s23031650

**Published:** 2023-02-02

**Authors:** Sabir Hossain, Xianke Lin

**Affiliations:** Faculty of Engineering and Applied Science, Ontario Tech University, Oshawa, ON L1G 0C5, Canada

**Keywords:** computer vision, depth perception, pseudo-LiDAR, self-supervised learning

## Abstract

Perception and localization are essential for autonomous delivery vehicles, mostly estimated from 3D LiDAR sensors due to their precise distance measurement capability. This paper presents a strategy to obtain a real-time pseudo point cloud from image sensors (cameras) instead of laser-based sensors (LiDARs). Previous studies (such as PSMNet-based point cloud generation) built the algorithm based on accuracy but failed to operate in real time as LiDAR. We propose an approach to use different depth estimators to obtain pseudo point clouds similar to LiDAR to achieve better performance. Moreover, the depth estimator has used stereo imagery data to achieve more accurate depth estimation as well as point cloud results. Our approach to generating depth maps outperforms other existing approaches on KITTI depth prediction while yielding point clouds significantly faster than other approaches as well. Additionally, the proposed approach is evaluated on the KITTI stereo benchmark, where it shows effectiveness in runtime.

## 1. Introduction

**Figure 1:**
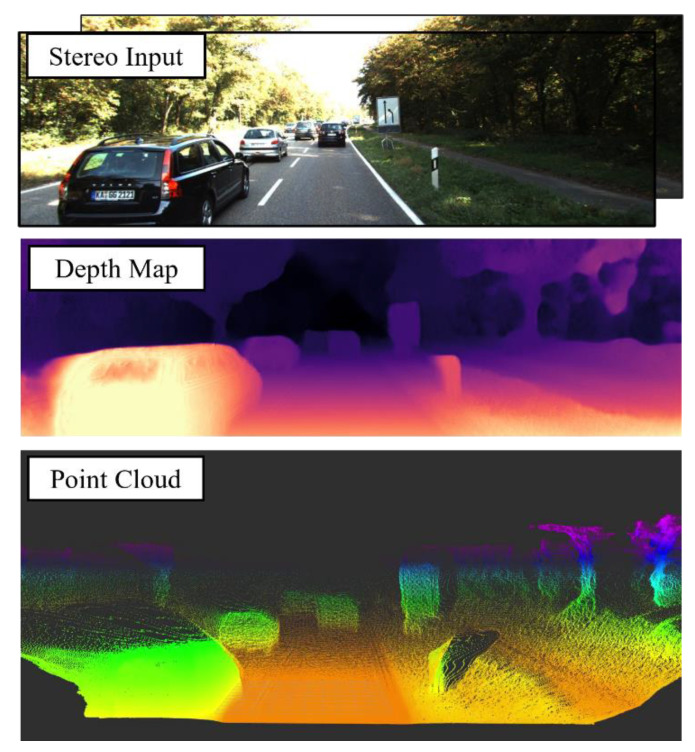
Point cloud generation from the depth map. (**Top image**): input to the pipeline-rectified stereo images from KITTI [4]; (**middle image**): estimated depth map using U-Net-based depth network; (**bottom image**): pseudo point cloud generation from the disparity.

Understanding the three-dimensional structure of the environment is possible for humans due to biological vision. Depth perception using computer vision technology is still one of the unsolved problems and most challenging issues in this research field. More significantly, proper depth perception is required for an autonomous system such as an autonomous delivery vehicle. It is possible to obtain such perception from the LiDAR point cloud; however, LiDAR is a very costly technology. It will drastically increase the production cost of a delivery robot system [1]. Without a doubt, a depth-predicting system is required to find an obstacle location and avoid a collision. Many researchers have already discussed the idea of alternative LiDAR solutions due to cost and over-dependency leading to safety risks. For example, the PSMNet model defined in the pseudo-LiDAR paper [2] is an image-based approach. The model architecture is too heavy, requiring more time to produce depth estimation. Therefore, the corresponding point cloud generation will be slower (average 1–6 Hz depending on the resolution) than LiDAR hardware (10 Hz). Our approach uses self-supervised stereo Monodepth2 [3] as the starting point and improved it to perform network training with stereo pairs in KITTI benchmark [4] datasets. Then, we used the generated disparity information to create the point cloud (shown in Figure 1). The main contributions of this paper are:

- Adopting a U-Net-based [5] encoder–decoder architecture as a depth network instead of the heavy PSMNet model to increase real-time performance
- Modifying the encoder network for the training step. The final result outperforms all the modes used by Monodepth2 [3] in terms of depth prediction.

It might be challenging to achieve a good balance between precision and latency. In order to solve this significant issue, this work suggests an optimal approach using self-supervised learning. In-depth experiments are also carried out by the authors to verify the proposed answer in comparison to earlier research. To evaluate our claim, we used a similar evaluation benchmark and produced a result that shows superior performance of depth estimation. Additionally, the result is compared on KITTI stereo matching benchmark. Then, we used the model to generate the point cloud and calculated the processing time it took to generate the depth map. Clearly, the results show that the approaches we took provide sufficient FPS to execute the whole operation in real time. Related works, methods, results, and conclusions are presented in Section 2, Section 3, Section 4 and Section 5, respectively.

## 2. Related Works

Image-based depth estimation to perform perception or localization tasks can be achieved using monocular vision [6] or stereo vision [2]. An algorithm such as DORN [7] achieves lower depth estimation errors than other previous works on monocular depth estimation [8,9,10]. On the other hand, stereo-based depth prediction systems [2] show more precision in estimating disparity. However, a promising solution requires a real-time operating speed with more efficiency. The BTS architecture [11] for depth estimation has a guided local planner layer in the decoding network. The method outperformed some of the evaluation metrics. However, the work does not provide the computational processing time against generating point cloud formation. Since the base networks have 49.5 million or more parameters, the network (ResNet50 or others) is very computationally expensive. In contrast to our proposed encoder module, the trainable parameters are around five times higher.

Recent studies are leveraging deep neural networks to learn model priors using pictorial depth, such as texture density or object perspective, directly from the training data [12]. Several technical breakthroughs in the past few years have made it possible to improve depth estimation based on the ground truth depth dataset. If ground truth depth is not available, a possible alternative is to train models using image reconstruction. Here, the model is fed either monocular temporal frames or stereo pairs of images as input. The model is trained by reducing error in image reconstruction by imitating the depth and projecting it into a nearby view. Stereo pair is one form of self-supervision. Since stereo pair of data is available and easy to obtain, a deep network can be trained to perform depth estimation using synchronized stereo pairs during training. For the problem of novel view synthesis, the authors proposed a model with discretized depth [13] and a model predicting continuous disparity [6]. Several advancements have also occurred in stereo-based approaches, including generative adversarial networks [14] and supervised datasets [15]. Moreover, there are approaches to predicting depth with minimized photometric reprojection error with use of relative pose from a source image with respect to a target image [3]. In their stereo approach, the authors used stereo pairs to calculate losses; however, the neural architecture does not obtain features of other image pairs. 

A stereo group-wise correlation method [16] computes the cost volume and divides the left and right features into groups along the channel dimension. Each group’s correlation maps are calculated to obtain several matching cost suggestions packaged into a cost volume. X. Guo et al. proposed an improved 3D stacked hourglass network to reduce the computation cost [16]. The RAFT-Stereo [17] architecture employs multi-level convolutional GRUs for accurate real-time inference and provides cutting-edge cross-dataset generalization results. CasStereoNet [18] presented a cost volume based on a feature pyramid encoding geometry and context at smaller scales to improve stereo matching, ranking first in the DTU benchmark [19] at the time of publication. A network based on Cascade and fused cost volume [20] is used to increase resilience of a stereo matching network by decreasing domain disparities and balancing the disparity distribution across datasets. StereoDRNet’s depth architecture [21] predicts view-constant disparity and occlusion maps, which aids the fusion system in producing geometrically consistent reconstructions. EPE (0.98) and FPS (4.3) outperform PSMNet [2]. LEAStereo [22], a deep stereo matching architecture, establishes an outperforming result on the KITTI [4,23,24] test dataset with fewer parameters and significantly shorter inference time. ACVNet [25], a stereo matching network, presents outperforming results in both quantitative and qualitative aspects. However, the runtime for these algorithms is very high.

We demonstrate that the existing depth estimation model can be adapted to generate higher-quality results by combining the stereo pair in input layers rather than using the pair to calculate relative pose loss only. Moreover, we used the modified model to generate point clouds in real time.

## 3. Method

**Figure 2:**
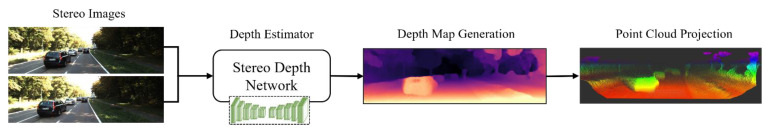
The proposed pipeline to generate pseudo-point-cloud-like LiDAR. From the stereo images, depth map prediction is completed using a modified stereo depth network, then back-projecting the pixel to 3D point coordinate system cloud generation from the depth map. (**Left image**): input to pipeline-rectified stereo images from KITTI [4]; (**middle image**): estimated depth map using U-Net-based depth network; (**right image**): pseudo point cloud generation from the disparity.

This section introduces the architecture of our modified deep network and then presents the strategy for splitting, point cloud generation, post-processing steps, and evaluation techniques used for comparison. The proposed pipeline is shown in Figure 2, and the modules are discussed in detail in this section. 

### 3.1. Stereo Training Using Depth Network

**Figure 3:**
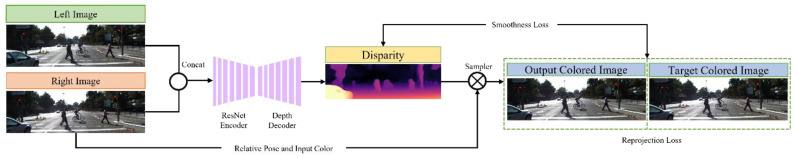
The modified approach with stereo pair in the encoder architecture. Losses are calculated based on the correct target frame from pair.

The proposed architecture is encoder–decoder-based classic U-Net (shown in Figure 3). The encoder is a pre-trained ResNet model [26], and the decoder converts the sigmoid output to a depth map. The primary network for training, U-Net architecture, merges various scale features with varying receptive field sizes and concatenates the feature maps after upsampling them by pooling them into distinct sizes. The ResNet encoder module usually accepts single RGB images as input. In the proposed method, the input is designed to take the image pair as input and provide estimation based on it. Therefore, the modified network works both for training and inference. The ResNet encoder is modified to accept a pair of stereo frames, or six channels, as input for the posture model. As a result, instead of the ResNet default of (3, 192, 640), the ResNet encoder uses convolutional weights in the first layer of shape (6, 192, 640). The depth decoder is a fully convolutional network that takes advantage of feature maps of different scales and concatenates them after upsampling. There is sigmoid activation at the last layer that outputs a normalized disparity map between 0 and 1. Table 1 shows the output total number of trainable parameters for encoder are 11,185,920 for (192,640) size of image input, whereas, for single-image-layer-based encoder, it would be 11,176,512. The ResNet encoder has 20 Conv2d layers, 20 BatchNom2D layers, 17 ReLU, 1 MaxPool2D layer, and eight basic blocks in total. The decoder layer has the same block, kernel size, and strides.

In monocular mode, Monodepth2 authors used temporal frame in Posenet [3] instead of stereo pair to calculate the extrinsic parameter of the camera and the pose of the image frame. Our approach will not rely on temporal frames for self-supervised prediction. The reprojection loss is calculated using SSIM [27] between prediction and target in stereo mode in stereo training. Metric reprojection error $L_{p}$ is calculated from relative pose $T_{s\to t}$ of source view denoted as $I_{s}$ with respect to its target image $I_{t}$. In our training, the other stereo pair will provide relative position $T_{s\to t}$ of source image $I_{s}.$ This rotation and translation information will be used to calculate mapping from the source frame to the target frame. Simultaneously, the ResNet architecture is fed with both image pairs (shown in Figure 3). The other can be considered the stereo pair of source images by considering one as the primary input. The target image is reprojected from the predicted depth and transformation matrix from the stereo pair using the intrinsic matrix. Then, the method used bilinear sampling to sample the source image from the target image. This loss aims to minimize the difference between the target picture and the reconstructed target image, in which depth is the most crucial factor. Instead of averaging the photometric error across all source frames, the method utilized the minimum at each pixel. The equation of photometric loss $L_{p}$ can be represented [3] as in the following Equation (1)

(1) $$L_{p}=\underset{\;s}{\min}RE\left(I_{t},\;I_{s\to t}\right)$$

Here, RE is the metric reconstruction error. $I_{t^{\prime }\to t}$ is obtained [28] from the projected depth D, intrinsic parameter K, and relative pose, as in the following Equation (2). 〈〉 is the bilinear sampling operator and prj() denotes 2D-cordinate of projected image.

(2) $$I_{s\to t}=I_{s}\langle prj\left(K,D,T_{s\to t}\right)\rangle$$

On the other hand, edge-aware smoothness loss $L_{s}\;$is also calculated between the target frame and mean-normalized inversed depth value. It boosts the model to recognize sharp edges and eliminate noises. The reprojection loss requires to have correct output image and target frame. Therefore, the method is designed to choose the proper target frame from the image pairs. The following Equation (3) is the final training loss function, which is the function used in [3]

(3) $$L_{\;}=µL_{p}+\lambda L_{s}$$

where µ is the mask pixel value, which is $µ\;\epsilon \;\left\{0,1\right\}$, obtained from the auto-masking method [3], and λ is the smoothness term, which is 0.001. Learning rate $10^{-4}$, batch size 12, epochs size 20 is used while training model size of both 640×192 and 1024×320. The edge-aware smoothness [3] can be described as following Equation (4)

(4) $$L_{s}=\left|\partial_{x}d_{t}^{*}\right|e^{-\left|\partial_{x}I_{t}\right|}+\left|\partial_{y}d_{t}^{*}\right|e^{-\left|\partial_{y}I_{t}\right|}$$

where $d_{t}^{*}=d_{t}^{\;}/\bar{d_{t}^{\;}}$ is the mean-normalized inverse depth [29] to discourage the estimated depth’s shrinking.

### 3.2. Dataset Splitting

We use the data split of Zhou et al. [28], which has 39,810 datasets for training and 4424 for validation. The intrinsic parameter provided by KITTI [4], which includes focal length and image center, is normalized with respect to image resolution. A horizontal translation of fixed size is applied to the horizontal transformation between stereo frames. The neural network is fed the image from the split file along with the corresponding pair. However, the rest of the calculation is based on the first taken from the split dataset, not the pair image. In stereo supervision, median scaling is not needed as the camera baseline can be used as a reference for scale.

### 3.3. Point Cloud Back-Projection

The depth (*z*) can be obtained from a stereo disparity estimation system that requires pair of right–left images with a horizontal baseline *b*. The depth estimation system will consider the left image as a reference and save the disparity map *d* with respect to the right image for each pixel $\left(x,y\right)$. Considering the focal length of the camera, *f*, the following Equation (5) of depth transformation can be obtained,

(5) $$z\left(x,y\right)=\frac{b\times f}{d\left(x,y\right)}$$

Point clouds have their own 3D coordinate with respect to a reference viewpoint and direction. Such 3D coordinate can be obtained by back-projecting all the depth pixels to a 3-dimensional coordinate system that will contain the point coordinates as ${\left[\left(X_{n},Y_{n},Z_{n}\right)\right]}_{n=0}^{N}$; *N* is the number of total points generated from the depth pixel. The back-projection was performed on the KITTI dataset images using their project matrices. The 3D location of each point can be obtained using the following Equations (6)–(8) with respect to the left camera frame reference, which can be calculated from the calibration matrices.

(6) $$width,\;X\left(x,y\right)=\frac{z\times \left(x-c_{x}\right)}{f_{x}}$$

(7) $$height,\;Y\left(x,y\right)=\frac{z\times \left(y-c_{y}\right)}{f_{y}}$$

(8) $$depth,\;Z\left(x,y\right)=z$$

where f is the focal length of the camera and $\left(c_{x},c_{y}\right)$ is the center pixel location of the image. Similar steps of back-projection are used to generate a pseudo-LiDAR point cloud [30]. 

### 3.4. Post-Processing Step

The method can adopt a post-processing step while training to achieve a significant accurate result in the evolution benchmark step. This adaption does not have any significance on the actual method. It is presented to compare with similar benchmarks that adopted these post-processing steps. Due to augmentation in the post-processing steps, the model tends to improve the estimation result. In order to obtain the model with the post-processing step, the stereo network is trained with the images two times, flipped and un-flipped. A threshold parameter randomizes this flip feature during training. Therefore, the model can be prepared both with and without post-processing steps. The flip feature occurs both in the image and its intrinsic parameters, including the baseline of the pairs. An unsupervised depth estimator introduces this type of two-forward pass-through network technique to improve the result [6]. 

### 3.5. Evaluation Metric

The evaluation benchmark primarily illustrated the errors between ground truth and prediction. The presented errors are mean absolute error (Abs Rel), squared error (Sq Rel), linear root mean squared error (RMSE), and logarithmic root mean squared error (RMSE log), respectively. These values indicate the lower, better result. On the other hand, δ < *x* denotes the ratio prediction and ground truth between *x* and 1/*x*. The results that are closer to 1 are better results. Instead of LiDAR reproject, ground truth depth from the KITTI depth prediction dataset [31] is used to evaluate the prediction method. During evaluation of our method, we used the same ground truth mentioned by Monodepth2 [3] while using stereo images as input in the encoder’s input layer. Moreover, KITTI stereo 2015 benchmark is also used for comparison.

## 4. Experiment and Results

**Figure 4:**
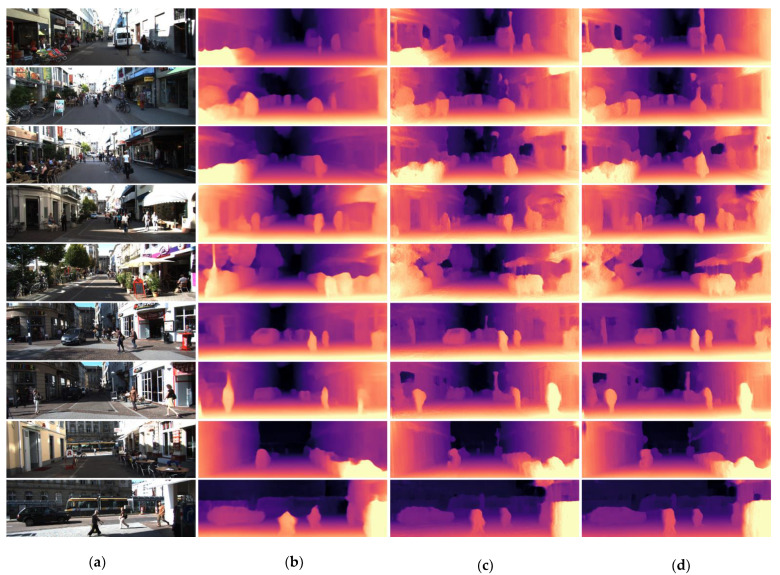
Qualitative results on the KITTI scene. (**a**) the primary image input; (**b**) generated result from the stereo depth estimator by Monodepth2 [3]; (**c**,**d**) are our results generated from 640 × 192 and 1024 × 320 models, respectively.

Figure 4 presents the qualitative results on a specific KITTI scene. The first-, second-, and seventh-row results show that our method adequately recognizes the pole. Moreover, other results show that size of pedestrians (for example, the result in row 9), shape of objects (for example, the results in rows 6 and 8), and buildings (for example, the results in rows 5 and 7) are more aligned with the original image. From this visual result, it is evident that our depth estimator can predict some of the features, such as poles or street signs, moving objects, and objects at far distances. Comparison is performed with other Monodepth2 modes: monocular only (M), stereo only (S), and both (MS), along with other self-supervised models presented in the paper [3]. Table 2 shows that our method (highlighted with bold font) outperforms all the variants, including self-supervised methods, except DORN [32]. Here, D refers to depth supervision, D* refers to auxiliary depth supervision, M refers to self-supervised mono supervision, S refers to self-supervised stereo supervision, and PP refers to post-processing. The other data were collected for comparison from [3]. The result achieving higher accuracy is due to introduction of stereo pairs in the input layer of ResNet architecture. Table 3 shows a comparison with the KITTI stereo benchmark since stereo pairs are introduced, which presents a satisfactory runtime for the proposed model. For stereo comparison, the disparity generated from the model is a scale with a scale faction and image width since the model was normalized to the image width. We used a common system to compare average processing speed (11th Gen Intel i7-11800H, 2.30 GHz, NVIDIA GeForce RTX 3070 Laptop GPU-8 GB, Ubuntu 18.04, Torch 1.10.0, CUDA 11.3). The Supplementary Materials section contains information to access the footage of the result. The results in Table 4 with ** show increase in FPS than other image resolutions. If the model resolution and image solution size are similar, the process does not use functional interpolation to resize the depth metrics to the image resolution. Therefore, it requires less time to predict the final output. Other resolutions present low FPS due to computationally expensive rescaling of the depth map. The processing time for PSMNet requires much longer than U-Net-based architecture. The overall steps to produce the point cloud are presented in Algorithm 1.
**Algorithm 1.** The overall steps: image to point cloud generation.**Input:** Image pair input**Output:** The 3D point cloud of the environment1:Initialize the encoder and decoder model2:Initialize the proper model and input size3:Initialize the calibration parameter, such as intrinsic and projection matrix.4:**while** image frames are available, **do**5:Read image pairs 6:Convert to torch tensor7:Concatenate the image pairs8:Extract the features using the encoder network9:Depth output using decoder network10:Functional interpolation of result **if** the size is different11:Squeeze the output to the array12:Project the disparity to points13:Convert to point field for visualization14:**end**

**Figure 5:**
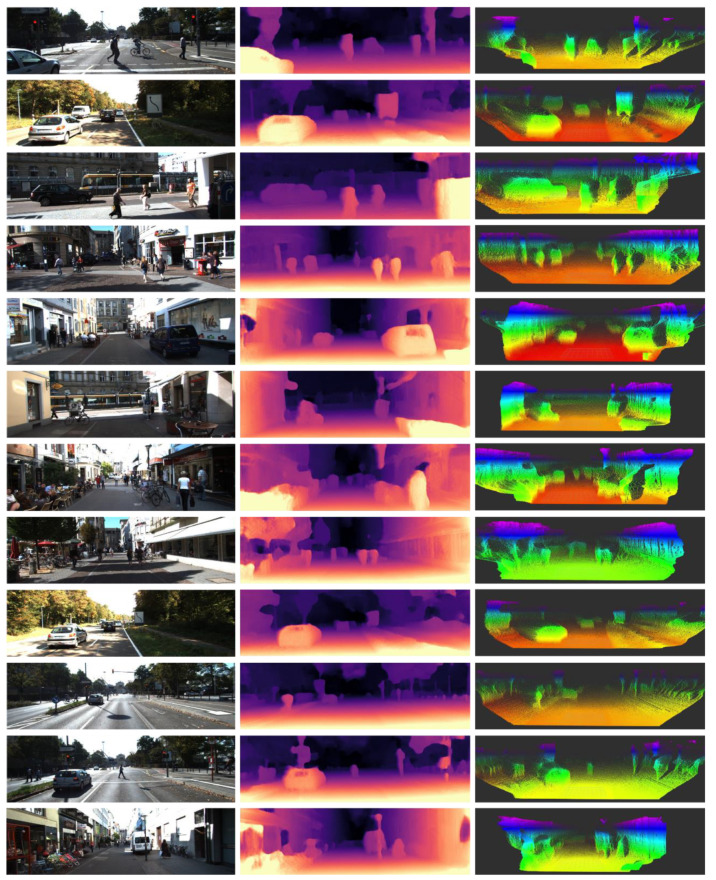
Visualization of point cloud generated from the depth estimation; the first column is the input pairs to the neural network, the second column is depth prediction, and the third column presents the point cloud result.

The main module responsible for FPS is depth prediction network. We presented the result with models 640 × 192 and 1024 × 320 in Table 4. The result includes processing of point cloud projection, whereas Table 3 provides only the runtime of depth estimation. Point cloud back-projection does not require extra time when the model architecture’s input size and the input image size are the same. Since it does not require using functional interpolation, the runtime for the whole algorithm is low and almost the same when the same model input and image input are used. Therefore, the computational process varies when the model input size and image input are different. Using stereo model 1024 × 320, we can obtain higher accuracy in real time. Figure 5 shows the point cloud visualization result on KITTI scenes. We used ROS converted bag of KITTI dataset and RViz to visualize the point cloud data. The point cloud visualization shows the perception of pedestrians, bicycles, cars, and traffic signs in 3D space.

## 5. Conclusions

In this paper, we have presented a strategy aimed at reducing the gap between point clouds from real LiDAR devices and image-based point clouds. We presented performance results for operating the model with U-Net architecture. Both versions of our resolution (average 57.2 and 31.9 FPS, respectively) indicate real-time operating performance. Moreover, we improved the network input layer by introducing stereo pairs to the input layer. Improvement in the stereo-based network is due to stereo information that helps the network to conceive more perceptions regarding moving and standstill objects. The final result shows more accurate results on the 1024 × 320 model and post-processing-based training on 1024 × 320. The improvement we achieved from the modified network is comparatively greater than its previous versions. Initially, we took a different approach to introduce more pixel features to the model. We tried to concatenate the temporal frames in the input layers; however, the result was poor. Later, we adopted the approach of stereo pairs since the model has no experience with stereo pairs. Since the work used stereo pairs, it is not technically suited for mono depth estimation. Due to use of stereo pairs, the computing process also increased. On the other hand, the LiDAR device is the most expensive commercial component for delivery vehicles. The image-based approach is the way to close this gap in cost. The proposed pipeline is entirely dependent on depth estimation’s accuracy, and poor depth estimation will result in erroneous point cloud data. Our approach aims to obtain an increase in FPS to generate fast pseudo-LiDAR. The model is trained with the KITTI raw dataset, which consists of 39,810 unrectified datasets for training and 4424 for validation. Therefore, it is not equally possible to compare the result with the KITTI stereo benchmark. However, the stereo benchmark is used for comparison with stereo matching algorithms, such as RAFT-3D [46], PSMNet [2], LEAStereo [22], CFNet [20], or ACVNet [25]. The result outperforms in terms of runtime. The outcome of this work does not raise any privacy issues or fairness issues. We do not believe our work or its possible applications pose any major concerns of security threats or human rights violations. In future work, we also aim to perform 3D object detection and SLAM algorithm over the point cloud achieved from depth prediction.

## Figures and Tables

**Table 1:** Model summary for encoder module.

Layer (Type: Depth-idx)	Output Shape	Param
Conv2D:1–1	[1, 64, 96, 320]	18,816
BatchNorm2d: 1–2	[1, 64, 96, 320]	128
ReLU: 1–3	[1, 64, 96, 320]	--
MaxPool2d: 1–4	[1, 64, 48, 160]	--
Sequential: 1–5	[1, 64, 48, 160]	--
BasicBlock: 2–1	[1, 64, 48, 160]	73,984
BasicBlock: 2–2	[1, 64, 48, 160]	73,984
Sequential: 1–6	[1, 128, 24, 80]	--
BasicBlock: 2–3	[1, 128, 24, 80]	230,144
BasicBlock: 2–4	[1, 128, 24, 80]	295,424
Sequential: 1–7	[1, 256, 12, 40]	--
BasicBlock: 2–5	[1, 256, 12, 40]	919,040
BasicBlock: 2–6	[1, 256, 12, 40]	1,180,672
Sequential: 1–8	[1, 512, 6, 20]	--
BasicBlock: 2–7	[1, 512, 6, 20]	3,673,088
BasicBlock: 2–8	[1, 512, 6, 20]	4,720,640
		Total params: 11,185,920 Trainable params: 11,185,920 Non-trainable params: 0

**Table 2:** Quantitative results with all variants of Monodepth2 [3] and other self-supervised methods on the KITTI 2015 dataset.

Method	Train Input	Abs Rel	Sq Rel	RMSE	RMSE log	δ < 1.25	δ < 1.25^2^	δ < 1.25^3^
AdaDepth 2018 [10]	D*	0.167	1.257	5.578	0.237	0.771	0.922	0.971
Kuznietsov 2017 [33]	DS	0.113	0.741	4.621	0.189	0.862	0.96	0.986
DVSO 2018 [34]	D*S	0.097	0.734	4.442	0.187	0.888	0.958	0.98
SVSM FT 2018 [15]	DS	0.094	0.626	4.252	0.177	0.891	0.965	0.984
Guo 2018 [32]	DS	0.096	0.641	4.095	0.168	0.892	0.967	0.986
DORN 2018 [35]	D	0.072	0.307	2.727	0.12	0.932	0.984	0.994
Zhou 2017 [28]	M	0.183	1.595	6.709	0.27	0.734	0.902	0.959
Yang 2018 [7]	M	0.182	1.481	6.501	0.267	0.725	0.906	0.963
Mahjourian 2018 [36]	M	0.163	1.24	6.22	0.25	0.762	0.916	0.968
GeoNet 2018 [37]	M	0.149	1.06	5.567	0.226	0.796	0.935	0.975
DDVO 2018 [29]	M	0.151	1.257	5.583	0.228	0.81	0.936	0.974
Ranjan 2019 [38]	M	0.148	1.149	5.464	0.226	0.815	0.935	0.973
EPC++ 2020 [39]	M	0.141	1.029	5.35	0.216	0.816	0.941	0.976
Struct2depth 2019 [40]	M	0.141	1.026	5.291	0.215	0.816	0.945	0.979
Monodepth2 w/o pretraining 2019 [3]	M	0.132	1.044	5.142	0.21	0.845	0.948	0.977
Monodepth2 (640 × 192), 2019 [3]	M	0.115	0.903	4.863	0.193	0.877	0.959	0.981
Monodepth2 (1024 × 320), 2019 [3]	M	0.115	0.882	4.701	0.19	0.879	0.961	0.982
BTS ResNet50, 2019 [11]	M	0.061	0.261	2.834	0.099	0.954	0.992	0.998
Garg 2016 [41]	S	0.152	1.226	5.849	0.246	0.784	0.921	0.967
Monodepth R50 2017 [6]	S	0.133	1.142	5.533	0.23	0.83	0.936	0.97
StrAT 2018 [42]	S	0.128	1.019	5.403	0.227	0.827	0.935	0.971
3Net (VGG), 2018 [43]	S	0.119	1.201	5.888	0.208	0.844	0.941	0.978
SuperDepth & PP, 2019 [44] (1024 × 382)	S	0.112	0.875	4.958	0.207	0.852	0.947	0.977
Monodepth2 w/o pretraining 2019 [3]	S	0.13	1.144	5.485	0.232	0.831	0.932	0.968
Monodepth2 (640 × 192), 2019 [3]	S	0.109	0.873	4.96	0.209	0.864	0.948	0.975
Monodepth2 (1024 × 320) 2019 [3]	S	0.107	0.849	4.764	0.201	0.874	0.953	0.977
Monodepth2 w/o pretraining, 2019 [3]	MS	0.127	1.031	5.266	0.221	0.836	0.943	0.974
Monodepth2 (640 × 192), 2019 [3]	MS	0.106	0.818	4.75	0.196	0.874	0.957	0.979
Monodepth2 (1024 × 320), 2019 [3]	MS	0.106	0.806	4.63	0.193	0.876	0.958	0.98
**Ours w/o pretraining (640 × 192)**	**S**	**0.083**	**0.768**	**4.467**	**0.185**	**0.911**	**0.959**	**0.977**
**Ours (640 × 192)**	**S**	**0.080**	**0.747**	**4.346**	**0.181**	**0.918**	**0.961**	**0.978**
**Ours (1024 × 320)**	**S**	**0.077**	**0.723**	**4.233**	**0.179**	**0.922**	**0.961**	**0.978**
**Ours (1024 × 320) + PP**	**S**	**0.075**	**0.700**	**4.196**	**0.176**	**0.924**	**0.963**	**0.979**

**Table 3:** Comparison of stereo matching methods on the KITTI stereo 2015 benchmark.

Methods	NOC			Runtime (s)
	D1-bg (%)	D1-fg (%)	D1-All (%)	
SED [45]	24.67	39.95	27.19	0.68
Raft-3D [46]	1.34	3.11	1.63	2
Mono-SF [47]	13.72	26.36	15.81	41
LEAStereo [22]	1.29	2.65	1.51	0.3
ACVNet [25]	1.26	2.84	1.52	0.2
CFNet [20]	1.43	3.25	1.73	0.18
monoResMatch [48]	21.65	19.08	21.23	0.16
PBCP [49]	2.27	7.71	3.17	68
PSMNet [2]	1.38	3.45	1.72	0.41
Ours (1024 × 320)	7.00	12.53	7.84	0.03

**Table 4:** Average processing speed.

Method	Image Resolution	FPS (Avg.)
PSMNet [2,30]	720 × 480	2.857
	1080 × 720	1.298
	640 × 256	6.25
	1024 × 320	3.125
Stereo Depth Estimation, Monodepth2 (640 × 192) [3]	720 × 480	40.6
	1080 × 720	21.7
	640 × 192	60.9 **
	1024 × 320	42.9
Ours (640 × 192)	720 × 480	32.1
	1080 × 720	19.1
	**640 × 192**	57.2 **
	1024 × 320	34.2
Ours (1024 × 320)	720 × 480	23.1
	1080 × 720	15.64
	640 × 192	26.6
	**1024 × 320**	31.9 **

** indicates the leading FPS performance on specific image resolution from specific models.

## Data Availability

Not applicable.

## Supplementary Materials

The real-time result footage is available here (https://www.xianke-lin.com/research, accessed on 31 January 2022). For the paper’s code, contact the following email: xianke.lin@ontariotechu.ca.
